# An Efficient Method for Testing the Quality of Drinking-Water Filters Used for Home Necessities

**DOI:** 10.3390/ijerph19074085

**Published:** 2022-03-30

**Authors:** Horea-George Crișan, Florina Șerdean, Corina Bîrleanu, Marius Pustan, Oana-Adriana Crișan

**Affiliations:** 1 Mechanical Systems Engineering Department, Faculty of Industrial Engineering, Robotics and Production Management, Technical University of Cluj-Napoca, 400641 Cluj-Napoca, Romania; florina.rusu@omt.utcluj.ro (F.Ș.); corina.barleanu@omt.utcluj.ro (C.B.); marius.pustan@omt.utcluj.ro (M.P.); 2 Micro-Nano Systems Laboratory, Technical University of Cluj-Napoca, 400641 Cluj-Napoca, Romania; oana.crisan@staff.utcluj.ro

**Keywords:** drinking-water quality, water distribution network, drinking-water filters, water pollutants, laboratory experimental tests, microscopic analyses, statistical analysis, Global Utility and ELECTRE method

## Abstract

This paper presents research conducted in the direction of analyzing the efficiency of filters used for drinking water intended for domestic consumption, with effects on the water quality gained from the public distribution network. A basic method that uses accessible techniques, such as optical microscopy and tests that involve the use of existing products on the consumer market, was developed regarding the filtration capacities of the main filters existing on the market—a method that has advantages, such as speed and ease of application, a unitary character in obtaining samples, low costs, and high efficiency. The technique approached is that of microscopy, and the samples used were taken from the laboratory tests made on the mentioned filters, using a specific experimental stand designed to support laboratory tests by using chosen filter cartridges. The research results obtained were analyzed to make a classification from the perspective of filtration efficiency, in terms of using statistical analysis tools (mathematical models and methods processed in MATLAB software). Moreover, by using a certain type of application based on specific mathematical algorithms, which takes into account some influential factors with a decisive role on household consumers, it was aimed to identify the optimal filter element for acquisition and use in its own regime. The aim of the study was to identify the optimal filter cartridge from the perspective of quality–price ratio.

## 1. Introduction

Water is the main resource consumed by all living beings. Regarding human beings, it has a significant role to play in maintaining a high level of health in terms of the quality of drinking water consumed from various sources of supply (public distribution networks, natural springs, surface water, bottled water, etc.). Although there are several alternative sources of drinking water available in most parts of the world, each has its advantages and disadvantages. All countries with a medium and high degree of development provide drinking water through public distribution networks to which almost all citizens have access. However, the quality of drinking water consumed by people differs and depends on several factors, such as its source and degree of purity, the existence of sewage treatment and treatment plants, and the existence of modern equipment to ensure compliant physical–chemical parameters of the water managed by the supplying companies, of the efficient distribution, and of the transport logistics for the drinkable water, etc. [1,2,3,4].

About primary sources of drinking water, such as the direct use of surface run-off water, it can be said that they are underdeveloped areas isolated worldwide and require priority in the construction of drinking-water treatment and supply networks. According to Moldovan et al. [5], “the radon activity concentrations in surface waters is low, usually below 1 Bq/L, while in groundwater vary from 1 to 50 Bq/L for rock aquifers in sedimentary rocks, 10 to 300 Bq/L for wells dug in soil, and 100 to 50,000 Bq/L in crystalline rocks”. The use of water directly from underground sources is sustainable but has the major disadvantage of limited locations of existing natural springs, the effort of moving and transporting limited amounts of water, and lack of control over its purity [6]. On the other hand, the purchase and use of bottled drinking water has the disadvantage of a relatively high purchase price (compared to running water, and this also includes the need to travel for purchase and transport it), the need to store it in limited quantities, and last but not least, from an ecological perspective, PET (polypropylene terephthalate) packaging being a major source of environmental pollution. In a study conducted in 2018, led by Mason et al. [7], it was stated that from a total of 260 water bottles processed, approximately 93% showed some sign of microplastic contamination. This has a negative impact on human health and also on the environment [8,9]. Moreover, it was stated that by decreasing losses and increasing the recycle rate, the effect of global warming could be reduced by 14.5% and 18.9% respectively [10]. In these circumstances, it is clear that where it is viable, the water provided by public networks is consumed directly or in culinary preparations (without including the water needed for housekeeping), so it is imperative to focus on some effective methods of increasing the quality for this type of product [11].

In Romania or abroad, water quality is influenced by direct inputs, such as a factory or treatment plants, or by nutrients and pesticides from agriculture and pollutants released into the air by industry. Although in many areas, through government involvement and appropriate local or national policies or using their own sources, the companies responsible for providing drinking water have ensured a high degree of drinking-water quality at the treatment plant or at the distribution point, this quality often differs from that of water at the terminus point (place of consumption). It happens due to water contamination by several agents, including microbiological (microorganisms, microfauna developed inside the distribution pipes) or mechanical (particles resulting from metal oxidation or erosion, detached from the walls of the pipes, mineral sediments, etc.) ones. This fact can be explained in several ways, such as the age and condition of the drinking water distribution system, the type of materials used for existing pipes, the costs of replacing network elements (both financial and technical through large-scale works, which would require the cessation of supply, blocking of traffic arteries, long duration, and so on) [12,13,14,15].

Therefore, equipping and using end-to-end drinking-water-filtration systems is an effective solution for ensuring the high quality of drinking water consumed by household consumers.

In this context, the aim of this paper was, on the one hand, to test the filtering capacity regarding a series of household filters available on the consumer market. It was made possible by setting up an experimental stand to test the filters under similar conditions, using microscopic techniques to identify polluting impurities and statistical analysis tools to interpret the data obtained.

On the other hand, the aim was to identify the optimal filter element for the household consumer by using mathematical models based on a series of factors that have a major influence on household consumers [16,17,18]. The results of these analyses were followed by the application of optimization methods in decision making, which have the role of validating the results obtained and providing the necessary information based on which household consumers perceive the importance of water consumption with high-quality parameters, the existence of easy possibilities but also efficient and economical solutions to ensure this quality, and, based on scientific results, their ability to decide to invest optimally in the quality filtration–price ratio while achieving the high-quality level of drinking water consumed, a requirement of the proposed desideratum.

The innovative element consists of achieving a classification by identifying the position of the filters, from the perspective of the quality–price ratio, oriented in two directions: (1) high quality at the expense of price and (2) low price at the expense of quality. These results were obtained by running the mathematical models that were used.

The identification of the optimal filtering cartridge from the filtering capacity–purchase price ratio point of view presents interest due to the economic situation of Romania’s citizens. According, for example, to the “GfK Purchasing Power Europe 2020” study, Romania is 31st in the European rankings when it comes to average per capita purchasing power; more specifically, Romania is around 60% below the European average. Moreover, Romania also has a very large gap between rich and poor. Hence, there are rich people who would be interested only in the results of the water-quality filtration regardless of the filter price, while the poor people are highly interested in the ratio between the water-quality filtration–price to make an informed decision and save money, if possible, especially since the cartridges are consumables. Obviously, the poor people could not afford to buy more filters and take them to a laboratory to be analyzed. Therefore, the analysis encompassed in this research is performed for different weights of the two considered criteria (quality filtration and price) in order to cover the interests of the Romanian citizens with different purchasing power.

## 2. Materials and Methods

As the results of the research are addressed to the general public who consume drinking water supplied through public distribution networks in Romania, the main five types of filters for mechanical filtration of this water were analyzed. The selection criteria of the studied filter cartridges were based on (1)accessibility for the general public, and this criterion is met, as they are found on the market in the offer of large specialty stores, with the purchase price reflecting the easy possibility to be purchased and replaced as often as necessary, and (2) last but not least, the technical–functional characteristics about the most important construction and filtration aspects, such as the materials of which they are made or the filtration capacity and the service life.

**Figure 1:**
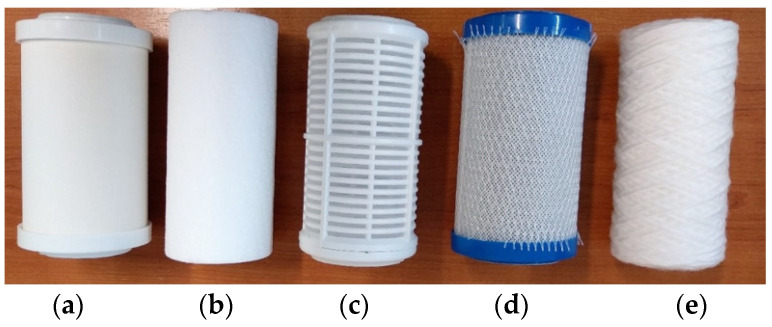
Analyzed filters: (**a**) Ceramic filter cartridge used for mechanical filtration of particles such as rust, sand, or other impurities, up to 0.3 µm. The maximum operating temperature is 30 °C. It can improve taste and smell from the water and has a resin component. (**b**) Polypropylene filter cartridge obtained by melting and expanding polypropylene; it can retain mechanical impurities (sand, rust, etc.) with the size up to 5 µm. The maximum operating temperature is 40 °C. (**c**) Washable filter cartridge retains impurities (sand, coal dust, sludge, rust, and other sediments) up to 70 µm. It has the advantage of reusing by washing. The maximum operating temperature is 70 °C. (**d**) Pressed activated carbon cartridge obtained by carbon-block technology; it can retain impurities or organic substances up to 1 µm and remove chlorine from the water (unpleasant taste and smell). The maximum operating temperature is 45 °C. It can improve taste and smell from the water but can also remove heavy metals. (**e**) Fabric filter cartridge made of polypropylene retains mechanical impurities (sand, rust, etc.) up to 25 µm. The maximum operating temperature is 45 °C. It can remove the bacteria and heavy metals from water. All have the same manufacturer, who is based in Poland, and they are imported in the European Union. All tested cartridges supported a water pressure of up to 8 (bar), but the test was performed at the mains pressure, with a constant value below this limit.

**Figure 2:**
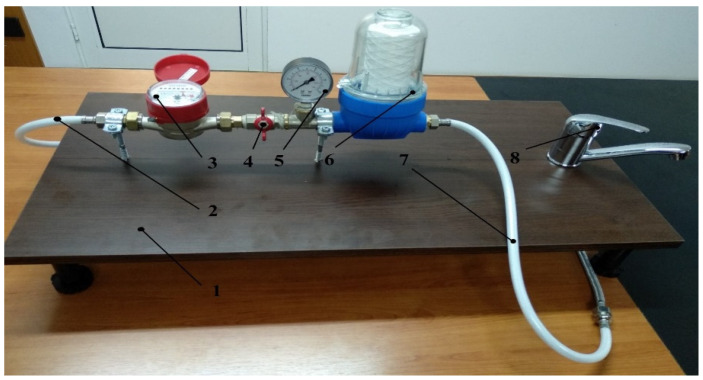
Experimental stand for testing the drinking water filters.

Therefore, the filters subject to analysis according to the material structure of the filter cartridge of which they are composed are named as follows (these are shown in Figure 1 [19], ordered according to the description and according to the manufacturers’ specifications):

The filter cartridges manufacturers’ prescriptions also indicate the recommendation to use these filter cartridges within 6 months from the date of installation or at a quantity of 3 m^3^ of drinking water consumed (passed through). To be able to test the five filter cartridges under similar conditions, until the maximum service life is reached, an experimental stand was developed, modeled, and built for this purpose (shown in Figure 2). The experimental stand consists of the following components: (1) support table with adjustable legs; (2) mains water inlet hose; (3) water-quantity meters; (4) water-pressure regulating valve; (5) water-pressure indicator; (6) drinking-water filter (consisting of filter housing and removable filter cartridge); (7) filtered water outlet hose; and (8) double-acting water tap (post-filtration flow regulation and post-filtration impurity retention on auxiliary support—absorbent paper).

The drinking-water supply company that was targeted for the experimental tests is “Someș Water Company S.A.” (Gherla, Romania) (see Supplementary Materials, Figure S2), and the test point, where the experimental stand was connected to the drinking water supply network is Gilău village, where the supplied drinking-water treatment plant is also located, and thus, the distance of water transport to the test point was considerably reduced. It should also be noted that this distribution company provides one of the highest-quality types of water nationwide [20,21]; however, its quality at the endpoint (end consumer) may suffer, and there is a real interest in improving it through efficient methods, such as the use of filter cartridges.

From a technical point of view, after connecting the stand to the water network, each filter cartridge was inserted into the housing intended for them on the experimental stand and through each one was passed the amount of 3 m^3^ of water. At the time of reaching the maximum service life recommended by the filter cartridges manufacturers, an equal amount of filtered water of 50 cm^3^ was collected. Using porous absorbent paper support with a density of 80 gr/m^2^, these were impregnated with the amount of water taken and filtered by the five tested cartridges (two samples on identical absorbent support).

**Figure 3:**
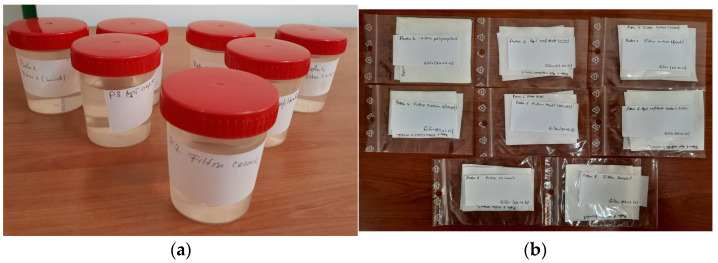
(**a**) Post-filtration water samples; (**b**) samples of impregnated filter paper.

Subsequently, they were allowed to dry in a room with controlled humidity and temperature, and after 5 h of the expected time to reach ambient conditions, the samples were isolated for analysis under a microscope. Figure 3a,b shows images of water samples taken after filtration, respectively, and of absorbent paper samples impregnated and prepared for microscopic analysis.

Samples of absorbent paper impregnated with post-filtration tested water were subjected to microscopic analysis to identify the type-size of mechanical deposits resulting from residual particles in the filtered water.

About the filtering capacity declared by the tested filter cartridge manufacturers, the microscopy technique used is optical. Thus, the Optika 10/0.25 microscope with magnification power ×100 was used to analyze the samples of absorbent paper impregnated with the tested water, and the computer program Cooling Tech was used for the scalar calibration and interpretation of the microscopic samples. The microscope is shown in Figure S1 (see Supplementary Materials).

After sampling and microscopic processing of the analyzed samples, the recorded results were interpreted from a statistical point of view. The methods were, on the one hand, used to determine a hierarchy of test cartridge test results in terms of the size and agglomeration density of the post-filtration residual particles in relation to the filtration capacity declared by the manufacturer. The Microsoft Excel program was used to compile this register, and the results are presented in tabular form. For interpretation of the results and to identify the efficiency of the filter cartridges in order to support household consumers to invest in the purchase of filter cartridges, the Kriging method was applied.

From the economic point of view, there are several reasons in favor of estimating the residual particle dimensions resulting after testing filters. The most important one is that testing a large range of filters is a time-consuming process, and usually, it involves significant costs. Therefore, estimating the dimensions of the residual particles is a viable option because consumers are not interested in exact values but want to have a general idea in order to make an informed decision.

The interpolation method used in this study for estimating the residual particle dimensions for the five filters is called Kriging, and it has been successfully used in several different applications [22,23,24]. When using this method, a surrogate model is built based on a predictor that uses the criterion of minimum mean squared for prediction errors. The weights given to the input values are not all equal, with the ones given to the data closer to the prediction point being larger. The efficiency of this interpolation comes from allowing correlated errors and adapting the used weights whenever a new prediction is necessary [25].

This interpolation technique was implemented in the MATLAB toolbox called DACE (Design and Analysis of Computer Experiments). This toolbox allows the users to choose between regression models with polynomials of orders 0, 1, or 2.

In addition, the correlation model can be one of the seven models included in Table 1.

The correlations are of the following form:

(1) $$C\left(\theta ,a,b\right)=\prod_{j=1}^{n}C_{j}\left(\theta ,a_{j}-b_{j}\right)$$

where a and b are two n-dimensional points, and θ is a n−dimensional parameter.

The spline correlation model is given as follows:

(2) $$S\left(\xi_{j}\right)=\{\begin{array}{cc} 1-15\xi_{j}+30\xi_{j}^{3}, & if\;0\leq \xi_{j}\leq 0.2\\ 1.25{\left(1-\xi_{j}\right)}^{3}, & if\;0.2<\xi_{j}<1\\ 0, & if\;\xi_{j}\geq 1 \end{array}$$

The first step in estimating the values of the residual particle dimensions resulting after using water filters is creating a file with the input data consisting of the values of the filter capacity for the five filters included in this research and the corresponding five values of the maximum residual particle dimension. After checking if the input data are correct, the software creates the Kriging surrogate model based on that data. The first step in creating the model consists of normalizing the data, and it is followed by computing the distances and the regression matrix. Once the surrogate model is built, the prediction of the value for the residual particle dimension for a filter with a different capacity (that has not been tested) can be made.

On the other hand, the aim was to determine the optimal filtering capacity–acquisition cost ratio in order to highlight the optimal filter cartridge from the mentioned perspective. This desideratum is of real interest in the influence of the decision of the purchase and use by domestic users of this type of product to the detriment of the use of bottled water, for example, in PET-type packaging and with much higher costs.

When the consumer is faced with a choice between existing water filters on the market, this can be done in relation to several criteria, such as the size of the residual particles and the price of the filter. The choice between the filters tested for this paper was made using two methods: the Global Utility method and the ELECTRE method. Last but not least, it should be noted that the results obtained by applying these two methods come in addition to the results obtained through microscopic analysis and by applying the Kriging method and also represents the validation of research on the proposed desideratum.

Therefore, both methods facilitate the choice of the optimal variant and constitute a logical support for anticipating the advantages of different possible modes of action [27]. The optimal variant in the case of the Utility Method is established according to different decisional criteria and importance coefficients, and the utility of a variant is calculated according to the economic consequence of a variant depending on a decisional criterion.

The information base is presented in a matrix in the form of Table 2.

First of all, the values ***a_ij_*** are converted in utilities ***u_ij_*** (which are recorded in a table similar to Table 2). Then, for each criterion **C_j_**, _j=1,…,n_, the minimum value ***a_j_*_min_** is determined and also the maximum value ***a_j_*_max_**. Within each criterion, utility 1 is given to the “best” value ***a_ij_*** and utility 0 to the lowest value. For the rest of the values, the utilities ***u_ij_*** are calculated:

- In the case of maximum criteria: $u_{ij}=\frac{a_{ij}-a_{j\min}}{a_{j\max}-a_{j\min}}$
- In the case of minimum criteria: $u_{ij}=\frac{a_{j\max}-a_{ij}}{a_{j\max}-a_{j\min}}$.

Subsequently, the global utility ***UG_i_*** is calculated for each decision variant **V_i_**:

(3) $$UG_{i}=\sum k_{j}u_{ij}$$

where, finally, the variant with the maximum global utility is chosen.

The ELECTRE method is based on choosing the optimal variant that outperforms the other variants by calculating the utilities for each decision optimization criterion, by assigning the importance coefficients, and by calculating the concordance and discordance coefficients [28].

First of all, the decisional variants and the afferent consequences are established by taking into account a set of criteria that condition their appearance. Then, the utilities are determined as in the method presented above. The next step is to establish the concordance indices between two decision variants, according to the formula:

(4) $$C\left(V_{g},V_{h}\right)=\frac{\sum k_{j}^{*}}{k_{1}+\ldots +k_{m}}$$

where $\sum k_{j}^{*}$ is the sum of the importance coefficients of the criteria for which the following restriction is observed: $U\left(V_{g}\right)\geq U\left(V_{h}\right)$.

Next, we proceed to the determination of the discordance indices with the formula:

(5) $$D\left(V_{g},V_{h}\right)=\{\begin{array}{c} 0,\;\;\;\;\;\;\;\mathrm{if}\;U\left(V_{g}\right)\geq U\left(V_{h}\right)\\ 1/\alpha \max\left|U\left(V_{g}\right)-U\left(V_{h}\right)\right|,\;\mathrm{otherwise} \end{array}$$

where α = maximum gap between minimum and maximum utility.

Finally, the classification of variants is based on the difference between the indices of concordance and those of discordance [29].

The two used methods, ELECTRE and Global Utility method, are both part of the group of multicriteria decision methods. ELECTRE is a more elaborated method whose first step consists of using the Global Utility method for computing the utility of each variant. The method does not stop there, having the advantage that it takes into account both the concordance and the discordance relations between the variants, therefore offering a better assessment of the progress of one variant over another. In this research, the results of the Global Utility method are presented due to the fact that they are computed as an intermediary result in the ELECTRE method. Furthermore, it is interesting to compare the results obtained with the two methods: the same filters occupy the first position, but some filters are the first choice for more combinations of price and filter capacity weights.

## 3. Results

### 3.1. Microscopic Analysis of the Samples Taken

Once the test cycle of the filters used on the experimental stand was completed, and the absorbent paper samples were impregnated with post-filtration water and then dried under identical conditions of temperature and humidity, these were subjected to type-dimensions analyses regarding the mechanical particles deposited after filtration [30].

**Figure 4:**
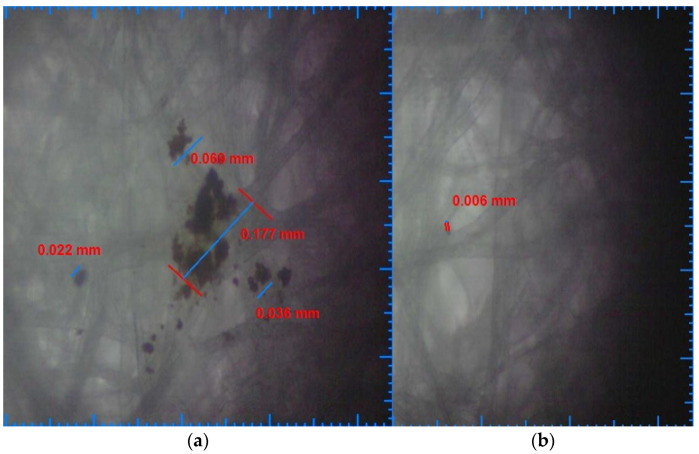
(**a**) Unfiltered water deposits sample; (**b**) ceramic filter water deposits sample.

As a benchmark in the development of the analysis of the sample, it started from the microscopy of a paper sample impregnated with unfiltered water (coming as a direct source) from the distribution network used and also as a supply for the experimental stand. Figure 4a shows the results obtained from this microscopic analysis. Residual particle deposits up to 0.177 mm was identified.

Furthermore, the order of the samples microscopic analysis was based on the filtering capacity of the tested filter cartridges. Therefore, Figure 4b shows the results of the microscopic analysis for the particles resulting from the water filtration with the ceramic filter cartridge. Residual particle deposits up to 0.006 mm were identified.

**Figure 5:**
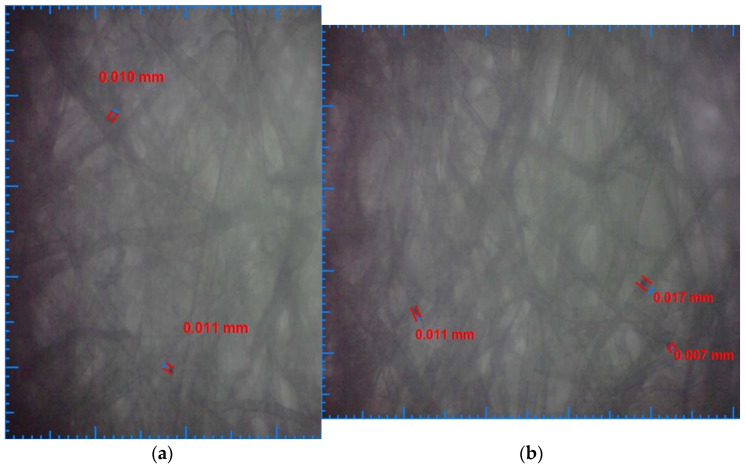
(**a**) Polypropylene filter water deposits sample; (**b**) activated carbon filter water deposits sample.

Figure 5a shows the results of microscopic analyses for deposited particles filtered by the polypropylene filter cartridge, and Figure 5b shows the results of microscopic analysis of particles resulting from water filtration with the activated carbon filter cartridge. It was observed that there were residual particle deposits up to 0.011 mm in the case of water filtered with a polypropylene filter cartridge and with dimensions up to 0.017 mm in the case of filtered water with an activated carbon filter cartridge.

**Figure 6:**
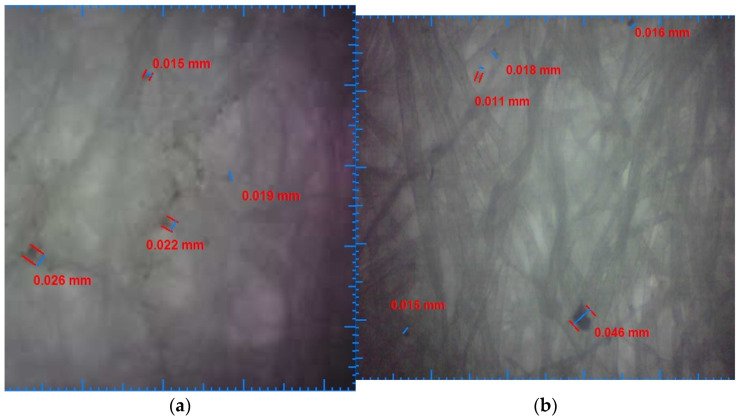
(**a**) Fabric filter water deposits sample; (**b**) washable filter water deposits sample.

Figure 6a shows the results of microscopic analysis for particle deposits from filtered water by the fabric filter cartridge, and Figure 6b shows the microscopic analysis results of particle deposits resulting from filtered water with washable filter cartridge. It was observed residual particle deposits up to 0.026 mm in the case of water filtered with the fabric filter cartridge and with dimensions up to 0.046 mm in the case of water filtered with the washable filter cartridge.

### 3.2. Statistical Interpretation of the Obtained Results

In order to make possible the implementation of the statistical analysis methods that allow the achievement of the desideratum consisting in establishing a hierarchy based on the results of the filter cartridge tests and microscopic analyses in terms of the size and agglomeration density of the post-filtration residual particles in relation to the filtration capacity declared by the manufacturer (by applying mathematical regression and correlation models), and respectively, the identification of the optimal filtering cartridge from the filtering capacity–purchase price ratio (using the decision support methods), the processed data were centralized and are presented in Table 3.

To estimate the residual particle dimensions resulting after using water filters, the software developed based on the Kriging interpolation method was used as follows:

*Call*: val = KrigingTest (x);

*Input*: x = the filter capacity required to be evaluated;

*Output*: val = predicted value of the residual particle dimension.

**Figure 7:**
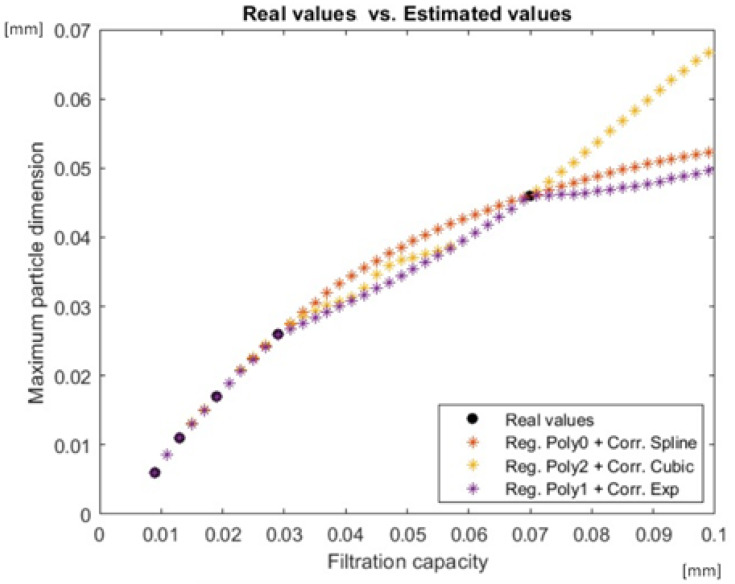
Estimated values using a model with a polynomial regressions and correlation models.

Before actually building the Kriging model and using it to estimate the dimensions of the residual particles of untested filters, the correlation and regression models proper for the given input data set had to be chosen. Thus, filters 2, 3, and 4 were, one at a time, eliminated from the input data set, being considered as test points, while the others were used as input data. Tests were performed for all possible combinations between the correlation and regression models. The best estimation with an error of 3.65% was obtained when using the regression model with a polynomial of order 0 and spline correlation model. For this combination, the estimated values for the residual particles for filters with the capacity 0.009 and 0.1 were computed using the surrogate Kriging model, and the results are encompassed in Figure 7 (plotted in red line). The smallest error of 3.99% using a regression model with a polynomial of order 2 was obtained for the cubic correlation model, and for this combination, the estimated values are plotted with yellow line. The smallest error of 5.5% using a regression model with a polynomial of order 1 was obtained for the exponential correlation model, and the estimated values for the residual particle dimensions are presented in purple line.

**Figure 8:**
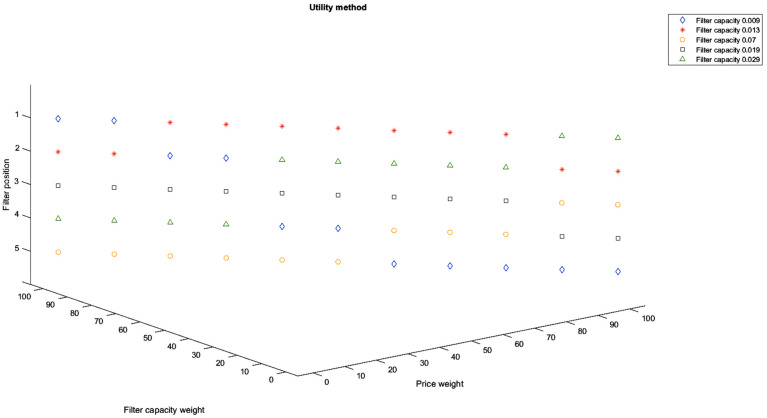
Filter classification with respect to different criteria weight combinations using the Global Utility method.

**Figure 9:**
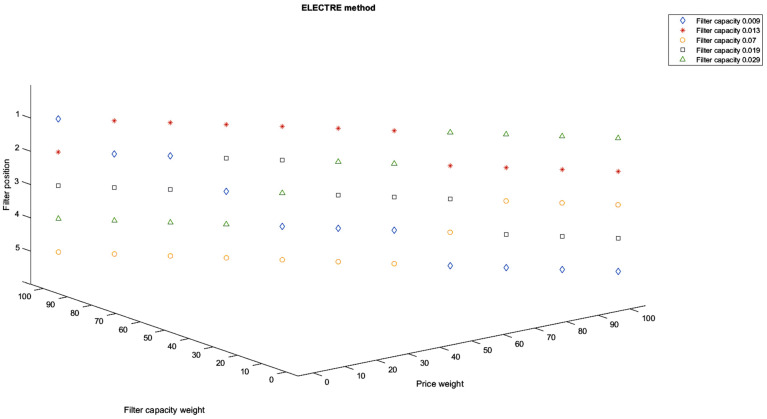
Filter classification with respect to different criteria weight combinations using the ELECTRE method.

Another aspect that presents interest is choosing the best filter between the ones available to consumers but concerning more criteria and not only the filter capacity. Obviously, each criterion might have a different weight for each consumer, and therefore, it would lead to different choices. The classification of the tested filter options was performed for all weight combinations (*k_d_*, *k_p_*), where *k_d_* is the weight of the dimensions of the residual particles, and *k_p_* is the weight of the price, $k_{d},k_{p}\in \left[0,100\right]$, $k_{d}+k_{p}=100$. The prices of the filters are included in Table 3. Using these prices as well as the residual particle dimensions determined experimentally for each tested filter, the results are presented in Figure 8 using the Global Utility method and in Figure 9 using the ELECTRE method for each possible combination of weights (considering a step of 10%).

As it can be seen in Figure 7, the estimated values of the maximum particle dimension increase with respect to the increase in the filtration capacity, as expected. However, the increase is associated with slope changes in the graph of the estimated values when the estimation is performed using the regression model with a polynomial of order 2 and cubic correlation model and using the regression model with a polynomial of order 1 and exponential correlation model.

As it can be seen in Figure 8 and Figure 9, the best filter options when also considering price are the ones with a filter capacity of 0.009 (ceramic), 0.013 (polypropylene), and 0.029 (fabric). The one with a filter capacity of 0.009 (ceramic) is the best option only when the price weight is 0 for the ELECTRE method or when the price weight is 0% and 10% for the Global Utility method. Then, for both methods, the position of this filter in the consumer preference drops as the price weight increases. For price weights between 10% and 60% for the ELECTRE method and between 20% and 80% for the Global Utility method, the preferred filter is the one with a filter capacity of 0.013 (polypropylene).

As the price weight increases over 60% for the ELECTRE method and over 90% for the Global Utility method, the best option is the filter with the capacity of 0.029 (fabric).

## 4. Conclusions and Discussion

To achieve the main objective of the research, more exactly the identification of the optimal filter in terms of efficiency and cost, we tested the main types of filter cartridges used for drinking water provided from the public network [31].

For this, several steps were followed: An experimental stand was developed, which allowed the testing of the five types of filters tested under similar conditions. After the end of the tests, samples of filtered water were taken and used for soaking parchment supports, which, after drying under ideal conditions, were subjected to microscopic analysis in order to identify the type-size of residual deposits after filtration. The results of the microscopic analyzes led to the possibility of a classification that was the basis for the application of the mathematical calculation methods to identify the optimal quality–price ratio regarding tested filters in terms of filtration efficiency. First, the Kriging method was used to estimate the residual particle dimensions resulting after using water filters, and in order to make it possible to apply, mathematical correlation and regression models were previously used, aiming to remove from the analysis filters that did not meet the necessary criteria. The results showed that the best estimation with an error of 3.65% was obtained when using the regression model with a polynomial of order 0 and spline correlation model, so the estimated values for the residual particles for filters with the capacity 0.009 and 0.1 were computed using the surrogate Kriging. The smallest error of 3.99% using a regression model with a polynomial of order 2 was obtained for the cubic correlation model and for this combination, and the smallest error 5.5% using a regression model with polynomial of order 1 was obtained for the exponential correlation model.

The classification of the tested filter options was performed for all weight combinations between the dimensions of the residual particles and the weight of the price. To make this possible, two methods were applied, the results of which were mutually validated as expected.

Both Global Utility and ELECTRE methods reflected the conclusion that the best filters options when also considering the price, although at a low level, are the ones with a filter capacity of 0.009 (ceramic), 0.013 (polypropylene), and 0.029 (fabric). When the share of the price increases, the filter capacity of 0.009 (ceramic) is downgraded to the detriment of the filter with a capacity of 0.013 (polypropylene) when using the Global Utility method and of the filter with a capacity of 0.029 (fabric) when using ELECTRE method.

Future directions for expanding the research could be to test and analyze a wider range of filter elements, possibly with intermediate filtering capabilities and different prices and lifetimes, but also using drinking water from another source of public distribution although it should be noted that this research has reflected the existence of significant residual particle deposits even if the water from the source used is purified and treated, and it is assumed that this product is of the highest quality provided (in terms of physical and chemical parameters measured as being public data) but also that the distribution network would support this quality. Another direction of research development may be the performance of pre-filtration and post-filtration drinking-water microbiology tests by the method of direct, closed-cell testing using cellular liquid analysis modules (for example, using “Universal Liquid Cell-Park Systems” as a modular part of an atomic force microscope-AFM).

## Figures and Tables

**Table 1:** Correlation models [26].

Name	*C_j_ (θ, d_j_)*, where *d_j_* = *a_j_ − b_j_*
EXP	exp(−*θ_j_ |d_j_*|)
EXPG	exp(−*θ_j_* |*d_j_*|*^θn^*^+1^), where 0 < *θ_n_*_+1_ ≤ 2
GAUSS	exp(−*θ_j_ d_j_*^2^)
LIN	max{0, 1 − *θ_j_* |*d_j_*|}
SPHERICAL	1 − 1.5*ξ_j_* + 0.5*ξ_j_*^3^, where *ξ_j_* = min{1, *θ_j_* |*d_j_*|}
CUBIC	1 − 3*ξ_j_*^2^ +2*ξ_j_*^3^, where *ξ_j_* = min{1, *θ_j_ |d_j_*|}
SPLINE	S(*ξ_j_*), where *ξ_j_* = *θ_j_ |d_j_*|

**Table 2:** Matrix of values.

Criteria	c_1_	c_2_	…	c_j_	…	c_n_
**Coefficients of importance**	k_1_	k_2_	…	k_j_	…	k_n_
**Variants**						
**V_1_**	a_11_	a_12_	…	a_1j_	…	a_1n_
**V_2_**	a_21_	a_22_	…	a_2j_	…	a_2n_
**…**			…		…	
**V_i_**	a_i1_	a_i2_	…	a_ij_	…	a_in_
**…**			…		…	
**V_m_**	a_m1_	a_m2_	…	a_mj_	…	a_mn_

**Table 3:** Centralized data on filtering capacity, tests and analyses results, and the purchase price.

	Filter Type and Filtration Size (Indicated by the Manufacturer) (mm)											
	Ceramic		Polypropylene		Washable		Pressed Activated Carbon		Fabric		Unfiltered Test Sample	
	0.0003–0.009		0.005–0.013		0.02–0.07		0.01–0.019		0.025–0.029		1	
**Number of particles identified**	1		2		5		3		4		4	
**Linear particle size (average)**	**Average**	**Maximum**	**Average**	**Maximum**	**Average**	**Maximum**	**Average**	**Maximum**	**Average**	**Maximum**	**Average**	**Maximum**
**(mm)**	0.006	0.006	0.0105	0.011	0.021	0.046	0.017	0.017	0.02	0.026	0.076	0.177
**Purchase price (lei/filter cartridge)**	25		7		12		15		6			

## Data Availability

Not applicable.

## Supplementary Materials

The following supporting information can be downloaded at: https://www.mdpi.com/article/10.3390/ijerph19074085/s1, Figure S1: Optical Microscope 10/0.25.; Figure S2: Map of the study area. Reference [31] is also cited in the supplementary materials.

## References

[1] Grafton R.Q., Pittock J., Williams J., Jiang Q., Possingham H., Quiggin J. (2014). Water Planning and Hydro-Climatic Change in the Murray-Darling Basin, Australia. AMBIO.

[2] Bisung E., Dickin S. (2020). Water Security. International Encyclopedia of Human Geography.

[3] Jones E., Wright J. (2011). Water Consumption and Implications for Exposure Assessment. Encyclopedia of Environmental Health.

[4] Amebelu A., Ban R., Bhagwan J., Brown J., Chilengi R., Chandler C., Colford J.M., Cumming O., Curtis V., Evans B.E. (2021). The Lancet Commission on water, sanitation and hygiene, and health. Lancet.

[5] Moldovan O.T., Skoglund R.Ø., Banciu H.L., Cucoș A.D., Levei E.A., Perșoiu A., Lauritzen S.-E. (2019). Monitoring and risk assessment for groundwater sources in rural communities of Romania (GROUNDWATERISK). Res. Ideas Outcomes.

[6] Damian G.E., Micle V., Sur I.M., Chirila Babau A.M. (2019). From environmental ethics to sustainable decision making: Assessment of potential ecological risk in soils around abandoned mining areas-case study “Larga de Sus mine” (Romania). J. Agric. Environ. Ethics.

[7] Mason S.A., Welch V.G., Neratko J. (2018). Synthetic Polymer Contamination in Bottled Water. Front. Chem..

[8] Dalhat M. (2021). Water resistance and characteristics of asphalt surfaces treated with micronized-recycled-polypropylene waste: Super-hydrophobicity. Constr. Build. Mater..

[9] Prabakaran E., Vijayakumar A., Rooby J., Nithya M. (2021). A comparative study of polypropylene fiber reinforced concrete for various mix grades with magnetized water. Mater. Today Proc..

[10] Kouloumpis V., Pell R.S., Correa-Cano M.E., Yan X. (2020). Potential trade-offs between eliminating plastics and mitigating climate change: An LCA perspective on Polyethylene Terephthalate (PET) bottles in Cornwall. Sci. Total Environ..

[11] Blkoor S.O., Ismail I., Oseh J.O., Selleyitoreea S., Norddin M.M., Agi A., Gbadamosi A.O. (2021). Influence of polypropylene beads and sodium carbonate treated nanosilica in water-based muds for cuttings transport. J. Pet. Sci. Eng..

[12] Zhang K., Chang S., Fu Q., Sun X., Fan Y., Zhang M., Tu X., Qadeer A. (2021). Occurrence and risk assessment of volatile organic compounds in multiple drinking water sources in the Yangtze River Delta region, China. Ecotoxicol. Environ. Saf..

[13] Alim M.A., AliAshraf A.F.M., Rahman A., Tao Z., Roy R., Khan M.M., Shirin S. (2021). Experimental investigation of an integrated rainwater harvesting unit for drinking water production at the household level. J. Water Process. Eng..

[14] Pinar-Méndez A., Fernández S., Baquero D., Vilaró C., Galofré B., González S., Rodrigo-Torres L., Arahal D.R., Macián M.C., Ruvira M.A. (2021). Rapid and improved identification of drinking water bacteria using the Drinking Water Library, a dedicated MALDI-TOF MS database. Water Res..

[15] Zhai Y., Liu G., van der Meer W.G. (2021). One-step reverse osmosis based on riverbank filtration for future drinking water purification. Engineering.

[16] Shafiquzzaman M. (2021). Effect of pre-aeration on the removal of arsenic and iron from natural groundwater in household based ceramic filters. J. Environ. Manag..

[17] Afkhami A., Marotta M., Dixon D., Ternan N.G., Montoya-Jaramillo L.J., Hincapie M., Galeano L., Fernandez-Ibanez P., Dunlop P.S. (2021). Assessment of low-cost cartridge filters for implementation in household drinking water treatment systems. J. Water Process Eng..

[18] Maciel P.M.F., Fava N.D.M.N., Lamon A.W., Fernandez-Ibañez P., ByrneJ A., Sabogal-Paz L.P. (2021). Household water purification system comprising cartridge filtration, UVC disinfection and chlorination to treat turbid raw water. J. Water Process Eng..

[19] Crișan O.-A. (2020). Studies and Research on the Application of the Concept of Circular Economy in Water Management. Ph.D. Thesis.

[20] Someș Water Company S.A Laboratoarele de Analize ale Companiei de apa Somes. https://www.casomes.ro/?page_id=1782.

[21] Dettori M., Arghittu A., Deiana G., Castiglia P., Azara A. (2022). The revised European Directive 2020/2184 on the quality of water intended for human consumption. A step forward in risk assessment, consumer safety and informative communication. Environ. Res..

[22] Chang W.-Y., Dai X.-G., Chen H.-W. (2004). A Case Study of Geostatistical Interpolation in Meteorological Fields. Chin. J. Geophys..

[23] Georgakarakos S., Kitsiou D. (2008). Mapping abundance distribution of small pelagic species applying hydroacoustics and Co-Kriging techniques. Essential Fish Habitat Mapping in the Mediterranean.

[24] Rusu F., Tudose L., Tudose C. (2015). Bearing life approximation using kriging. Acta Tech. Napoc.-Ser. Appl. Math. Mech. Eng..

[25] Van Beers W., Kleijnen J. Kriging Interpolation in Simulation: A Survey. Proceedings of the 2004 Winter Simulation Conference.

[26] Nielsen H.B., Lophaven S.N., Søndergaard J. DACE—A Matlab Kriging Toolbox. Computer Programme, Informatics and Mathematical Modelling, 2002, Technical University of Denmark, DTU. http://www2.imm.dtu.dk/pubdb/p.php?1460.

[27] Gâf-Deac M. (2002). Management modern. Elemente de Baza si Studii de Caz.

[28] Roy B. (1968). Classement et choix en présence de points de vue multiples. Rev. Fr. D’inform. Rech. Opér..

[29] Elaborarea Deciziilor. http://www.mpt.upt.ro/doc/curs/gp/Bazele_Managementului/Elaborarea_deciziilor_cap7.pdf.

[30] Börger T., Campbell D., White M.P., Elliott L.R., Fleming L.E., Garrett J.K., Hattam C., Hynes S., Lankia T., Taylor T. (2021). The value of blue-space recreation and perceived water quality across Europe: A contingent behaviour study. Sci. Total Environ..

[31] Map of the Study Area. https://www.google.com/maps/place/Compania+de+Ap%C4%83+Some%C8%99/@46.2363607,21.7509944,6.69z/data=!4m5!3m4!1s0x47490c1862855555:0xf81b2e004666c9a9!8m2!3d46.7760585!4d23.6050224.

