# Supplementary material for: An Efficient Method for Testing the Quality of Drinking-Water Filters Used for Home Necessities
Source: Int J Environ Res Public Health. 2022 Mar 30;19(7):4085. doi: 10.3390/ijerph19074085 (PMC8998660; doi:10.3390/ijerph19074085)
Supplement: Supplementary file 1 [file ijerph-19-04085-s001.zip › Supplementary Materials.pdf]

Supplementary Materials: The following are available online at www.mdpi.com/xxx/s1, Figure S1: Optical Microscope 10 / 0.25.; Figure S2: Map of the study area.

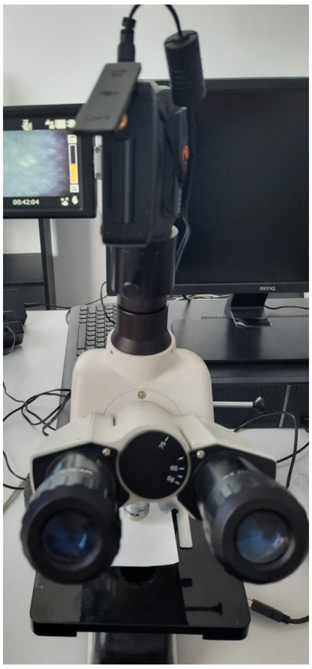

Figure S1. Optical Microscope 10 / 0.25.

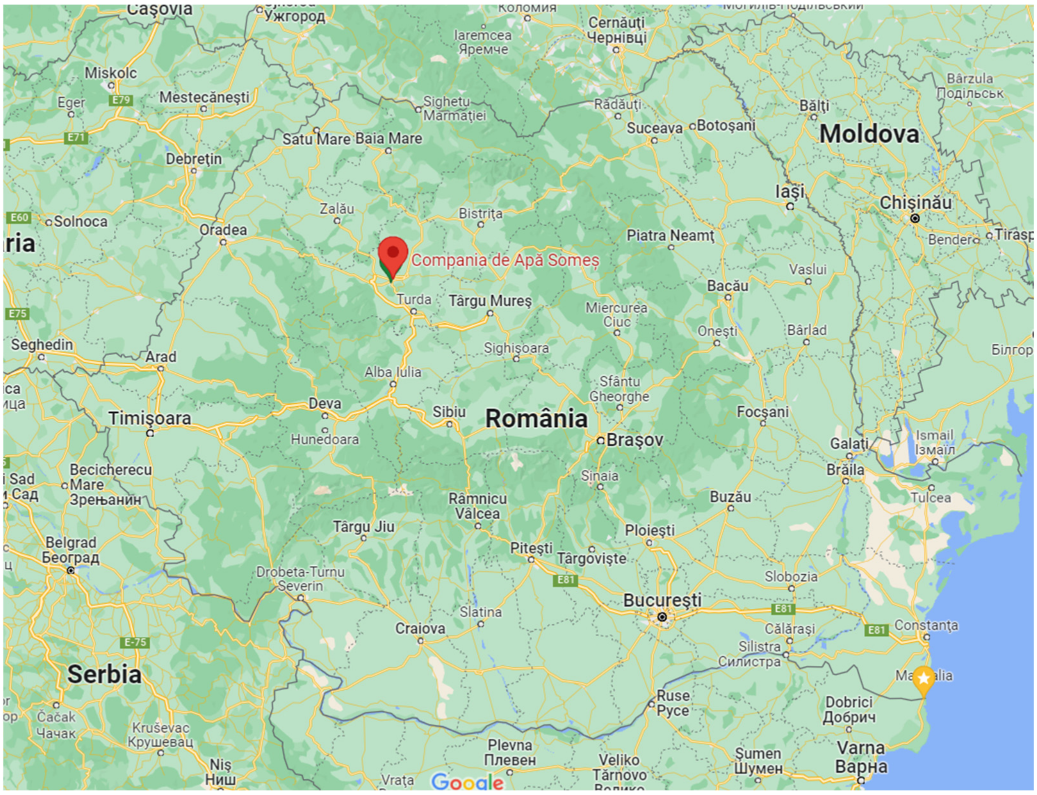

Figure S2. Map of the study area [31].

References

31. Map of the study area. Available online: https://www.google.com/maps/place/Compania+de+Ap%C4%83+Some%C8%99/@46.2363607,21.7509944,6.69z/data=!4m5!3m4!1s0x47490c1862855555:0xf81b2e004666c9a9!8m2!3d46.7760585!4d23.6050224.
