# Supplementary figures and images for: An Efficient Method for Testing the Quality of Drinking-Water Filters Used for Home Necessities
Source: Int J Environ Res Public Health. 2022 Mar 30;19(7):4085. doi: 10.3390/ijerph19074085 (PMC8998660; doi:10.3390/ijerph19074085)

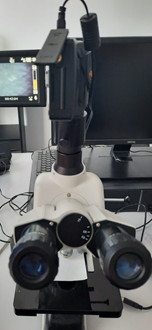

Supplement: Supplementary file 1 [file ijerph-19-04085-s001.zip › S1.png]

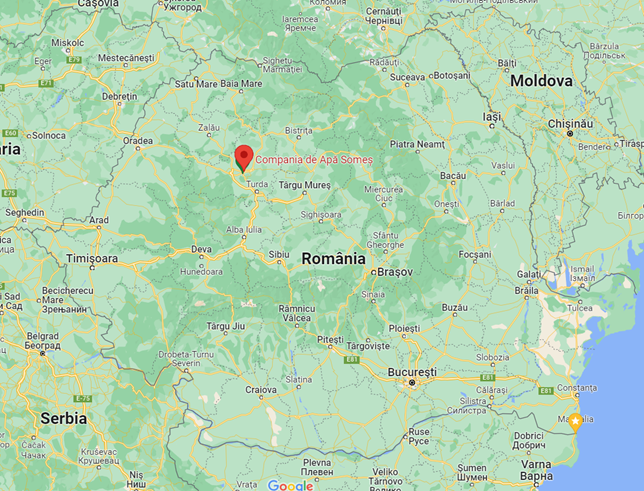

Supplement: Supplementary file 1 [file ijerph-19-04085-s001.zip › S2.png]
